# Plasma proteomic approach in patients with heart failure: insights into pathogenesis of disease progression and potential novel treatment targets

**DOI:** 10.1002/ejhf.1608

**Published:** 2019-11-06

**Authors:** Thong H. Cao, Donald J.L. Jones, Adriaan A. Voors, Paulene A. Quinn, Jatinderpal K. Sandhu, Daniel C.S. Chan, Helen M. Parry, Mohapradeep Mohan, Ify R. Mordi, Iziah E. Sama, Stefan D. Anker, John G. Cleland, Kenneth Dickstein, Gerasimos Filippatos, Hans L. Hillege, Marco Metra, Piotr Ponikowski, Nilesh J. Samani, Dirk J. Van Veldhuisen, Faiez Zannad, Chim C. Lang, Leong L. Ng

**Affiliations:** ^1^ Department of Cardiovascular Sciences University of Leicester and National Institute for Health Research Leicester Biomedical Research Centre, Glenfield Hospital Leicester UK; ^2^ Leicester Cancer Research Centre, Leicester Royal Infirmary University of Leicester Leicester UK; ^3^ Department of Cardiology University of Groningen Groningen The Netherlands; ^4^ Division of Molecular and Clinical Medicine, Ninewells Hospital and Medical School University of Dundee Dundee UK; ^5^ Division of Cardiology and Metabolism; Department of Cardiology (CVK) Center for Regenerative Therapies (BCRT); German Centre for Cardiovascular Research (DZHK) partner site Berlin; Charité Universitätsmedizin Berlin Berlin Germany; ^6^ Robertson Centre for Biostatistics Institute of Health and Wellbeing, University of Glasgow, Glasgow Royal Infirmary Glasgow UK; ^7^ University of Bergen, Stavanger University Hospital Stavanger Norway; ^8^ Department of Cardiology, Heart Failure Unit, Athens University Hospital Attikon, School of Medicine National and Kapodistrian University of Athens Athens Greece; ^9^ Institute of Cardiology, Department of Medical and Surgical Specialties, Radiological Sciences and Public Health University of Brescia Brescia Italy; ^10^ Department of Heart Diseases Wroclaw Medical University and Cardiology Department, Military Hospital Wroclaw Poland; ^11^ Inserm CIC 1433 Université de Lorraine Nancy France

**Keywords:** Heart failure, Mass spectrometry, Metabolism, Pathogenesis, Proteomics, Treatment target

## Abstract

**Aims:**

To provide insights into pathogenesis of disease progression and potential novel treatment targets for patients with heart failure by investigation of the plasma proteome using network analysis.

**Methods and results:**

The plasma proteome of 50 patients with heart failure who died or were rehospitalised were compared with 50 patients with heart failure, matched for age and sex, who did not have an event. Peptides were analysed on two‐dimensional liquid chromatography coupled to tandem mass spectrometry (2D LC ESI‐MS/MS) in high definition mode (HDMSE). We identified and quantified 3001 proteins, of which 51 were significantly up‐regulated and 46 down‐regulated with more than two‐fold expression changes in those who experienced death or rehospitalisation. Gene ontology enrichment analysis and protein–protein interaction networks of significant differentially expressed proteins discovered the central role of metabolic processes in clinical outcomes of patients with heart failure. The findings revealed that a cluster of proteins related to glutathione metabolism, arginine and proline metabolism, and pyruvate metabolism in the pathogenesis of poor outcome in patients with heart failure who died or were rehospitalised.

**Conclusions:**

Our findings show that in patients with heart failure who died or were rehospitalised, the glutathione, arginine and proline, and pyruvate pathways were activated. These pathways might be potential targets for therapies to improve poor outcomes in patients with heart failure.

## Introduction

Despite the latest advances in therapy for heart failure (HF), improving clinical outcomes remains a challenge.^1^, ^2^, ^3^, ^4^, ^5^, ^6^, ^7^ Whilst some patients with HF respond to appropriate therapy, many patients do not respond. The reason for the lack of response may be due to differences in clinical characteristics, underlying causes, biomarkers, genetic variants, and protein expression. Therefore, not all patients with HF achieve benefits from the same treatment.

Plasma is a desirably accessible window into the human proteome and its response to disease and therapy. However, analysis of plasma is extremely challenging because of the wide dynamic range of protein concentrations (10–12 orders of magnitude) and their structural complexity.^8^ Only 22 highly abundant proteins comprise 99% of the plasma proteome, so the challenge is to identify candidate biomarkers within the 1% of medium and low abundance proteins that are related to pathogenesis for clinical applications.^8^, ^9^, ^10^, ^11^, ^12^

Network analysis is a powerful tool for studying biological systems in health and disease^13^ that helps identify activated pathways, driven genes or mutations in the disease. In addition, network analysis can identify subtypes of the disease, classify patients as well as discover novel prognostic biomarkers and new targets for therapy that might support precision medicine. Therefore, using network analysis to compare the plasma proteome in HF patients with and without clinical events might not only provide insights into the pathogenesis of disease progression in HF, but might also identify new therapeutic targets. Our aim in this study was to provide the information about pathogenesis of disease progression and potential novel treatment targets in HF by investigation of the plasma proteome.

## Materials and methods

### Patient population

Overall, 100 patients with HF in this study were selected from the EU FP7 funded BIOSTAT‐CHF (A systems BIOlogy Study to TAilored Treatment in Chronic Heart Failure) project. This was a multicentre clinical study in Europe^14^ that aimed to identify poor outcomes in HF patients with standard treatment using a systems biology approach (including demographics, biomarkers, genetics, and proteomics).^15^, ^16^, ^17^, ^18^, ^19^, ^20^, ^21^, ^22^, ^23^, ^24^, ^25^, ^26^, ^27^ This project was conducted according to the Declaration of Helsinki that was approved by national and local ethics committees. All patients in this study had written informed consent. This study recruited 2516 patients who met inclusion and exclusion criteria according to the European Society of Cardiology guidelines.^28^ In brief, patients were >18 years old, presented symptoms of HF and had a left ventricular ejection fraction (LVEF) ≤40% and/or B‐type natriuretic peptide (BNP) >400 pg/mL or N‐terminal pro‐B‐type natriuretic peptide (NT‐proBNP) >2000 pg/mL as well as were not yet treated or treated with ≤50% of the target dose of both angiotensin‐converting enzyme inhibitor or beta‐blocker at the time of recruitment. Plasma samples were collected for proteomic analysis at the beginning of the project. The patients were treated with standard optimised therapy for HF, and drugs such as angiotensin‐converting enzyme inhibitors and beta‐blockers were up‐titrated up to 6 months. Patients were then followed up for clinical events such as death and rehospitalisation.^14^ There were two groups of patients with HF in this study that were sex‐ and age‐matched. Fifty patients with HF (25 male) who died or were rehospitalised were compared to 50 patients with HF (25 male) who did not have an event. The baseline patient characteristics are shown in *Table* 1.

**Table 1:** Patient characteristics in comparisons between the death/rehospitalisation heart failure group and the heart failure group with no events

Characteristics	Death/rehospitalisation (*n* = 50)	No events (*n* = 50)	*P*‐value
Age (years)	76.64 ± 8.14	76.64 ± 8.14	1.000
Male sex, *n* (%)	25 (50)	25 (50)	1.000
Clinical profile			
BMI (kg/m^2^)	30.01 ± 6.17	28.94 ± 6.66	0.471
Waist‐to‐hip ratio	1.01 ± 0.13	0.96 ± 0.10	0.018
NYHA class III/IV, *n* (%)	38 (76)	27 (54)	0.021
Systolic blood pressure (mmHg)	126.38 ± 20.63	130.94 ± 21.12	0.247
Diastolic blood pressure (mmHg)	66.92 ± 11.92	69.22 ± 12.24	0.324
Heart rate (bpm)	75.69 ± 19.91	73.94 ± 18.28	0.848
Outcome (death/rehospitalisation)	18/32	0/0	
Time to event (median, days)	121	NA	
LVEDD (mm)	56.46 ± 8.97	55.78 ± 10.94	0.736
LVESD (mm)	47.75 ± 14.03	45.09 ± 11.91	0.758
LVEF (median, %)	40	45	0.281
HFrEF/HFpEF, *n* (%)	22 (44.9)/18 (36.7)	20 (43.5)/21 (45.7)	0.503
Medical history, *n* (%)			
Hypertension	30 (60.0)	29 (59.2)	0.934
Myocardial infarction	30 (60.0)	24 (48.0)	0.229
PCI	12 (24.0)	5 (10.2)	0.069
CABG	18 (36.0)	6 (12.0)	0.005
Diabetes mellitus	21 (42.0)	13 (26.0)	0.091
Stroke	17 (34.0)	8 (16.0)	0.038
Atrial fibrillation	21 (42.0)	23 (46.9)	0.621
COPD	13 (26.0)	9 (18.0)	0.334
Peripheral arterial disease	21 (42.9)	8 (17.0)	0.006
Aetiology, *n* (%)			
Ischaemic heart disease	40 (80.0)	31 (67.4)	0.285
Non‐ischaemic heart disease	10 (20.0)	15 (32.6)	0.317
Laboratory			
Serum creatinine (μmol/L)	126.88 ± 58.56	107.16 ± 34.27	0.076
eGFR (mL/min^‐1^)	45.76 ± 14.23	51.34 ± 11.19	0.037
Haemoglobin (g/dL)	12.44 ± 1.72	12.99 ± 2.06	0.157
Red blood cell count (million/mm^3^)	4.26 ± 0.63	4.33 ± 0.69	0.357
White blood cell count (1000/mm^3^)	9.11 ± 3.10	7.97 ± 3.82	0.016
Platelet count (1000/mm^3^)	256.72 ± 87.65	232.58 ± 82.51	0.194
Glucose (mg/dL)	7.10 ± 2.14	7.37 ± 3.79	0.221
Albumin (g/L)	41.10 ± 5.30	42.88 ± 4.91	0.092
HDL cholesterol (mmol/L)	1.20 ± 0.45	1.24 ± 0.35	0.319
LDL cholesterol (mmol/L)	1.65 ± 0.76	1.99 ± 0.81	0.167
ALT (U/L)	26.04 ± 19.05	22.53 ± 11.38	0.590
AST (U/L)	27.34 ± 17.18	27.17 ± 14.30	0.719
Iron (g/dL)	12.00 ± 5.61	13.68 ± 6.04	0.141
Ferritin (ng/mL)	154.23 ± 192.84	146.94 ± 163.08	0.446
TSH (mU/L)	2.87 ± 2.42	2.33 ± 2.14	0.184
FT4 (pmol/L)	16.90 ± 4.12	17.37 ± 3.41	0.407
Sodium (mEq/L)	140.92 ± 3.83	139.76 ± 3.66	0.064
Potassium (mEq/L)	4.18 ± 0.47	4.30 ± 0.55	0.326
HbA1c (%)	6.58 ± 1.32	6.66 ± 1.48	0.822
NT‐proBNP (pg/mL)	6321.58 ± 7557.40	2616.38 ± 3442.63	0.003
Medication, *n* (%)			
ACE/ARB	30 (60.0)	38 (76.0)	0.086
Beta‐blocker	34 (68.0)	37 (74.0)	0.509
Aldosterone antagonist	14 (28.0)	14 (28.0)	1.000
Loop diuretic	48 (96.0)	47 (94.0)	0.646
Digoxin	6 (12.0)	11 (22.0)	0.183

ACE, angiotensin‐converting enzyme; ALT, alanine transaminase; ARB, angiotensin receptor blocker; AST, aspartate transaminase; BMI, body mass index; CABG, coronary artery bypass graft; COPD, chronic obstructive pulmonary disease; eGFR, estimated glomerular filtration rate; FT4, free thyroxine; HbA1c, glycated haemoglobin; HFpEF, heart failure with preserved ejection fraction; HFrEF, heart failure with reduced ejection fraction; HDL, high‐density lipoprotein; LDL, low‐density lipoprotein; LVEDD, left ventricular end‐diastolic diameter; LVEF, left ventricular ejection fraction; LVESD, left ventricular end‐systolic diameter; NT‐proBNP, N‐terminal pro‐B‐type natriuretic peptide; NYHA, New York Heart Association; PCI, percutaneous coronary intervention; TSH, thyroid stimulating hormone.

### Plasma sample collection and storage procedure

Blood samples of patients with HF were collected for proteomic work on admission to the study. Blood was obtained from supine patients after at least 15 min bed rest by venepuncture that was collected in 10 mL EDTA vacutainer tubes, inverted eight times and put on ice immediately. Plasma obtained after centrifugation at 1000 g for 15 min at 4°C was transferred to small aliquots and stored at −80°C until further analysis.

### Sample preparation

The greatest disadvantage of using mass spectrometry‐based proteomics is low throughput because of time‐consuming sample preparation and analysis on mass spectrometry and processing of proteomic data. Therefore, to reduce the sample preparation, sample analysis and data processing time, the plasma samples of patients with HF were pooled into two biological groups that were sex‐ and age‐matched. One group consisted of 50 patients with HF who died or were rehospitalised, and they were compared to the group of 50 HF patients who did not have an event. To do this, every plasma sample was thawed at room temperature and vortexed to ensure homogeneity. Then, a 100 μL aliquot of each plasma sample was taken and pooled to make two pooled plasma samples, including one pooled sample for HF patients with death/rehospitalisation and one pooled sample for HF patients without events.

Two pooled plasma samples were depleted of 14 high abundance proteins (including albumin, IgG, antitrypsin, IgA, transferrin, haptoglobin, fibrinogen, alpha 2 macroglobulin, alpha 1 acid glycoprotein, IgM, apolipoprotein A I, apolipoprotein A II, complement C3, and transthyretin) using a Multiple Affinity Removal System Human 14 (MARS 14, 4.6 × 100 mm, Agilent Technologies, Wilmington, DE, USA), exchanged buffers and concentrated. The samples were then reduced and alkylated before digestion with trypsin to peptides. One mg of each pooled sample was injected on a Gemini column to separate peptides (Gemini NX C18 110 Å, 150 × 2 mm, 3 μm particles, Phenomenex, Cheshire, UK) using a 110 min gradient in liquid chromatography. This step was performed offline on a high performance liquid chromatography (HPLC) system (Waters Corporation, Manchester, UK) which includes a Waters 600S controller, a Waters 486 Tunable Absorbance Detector and a Waters 626 Pump (Millipore, USA). Peptides were collected at every minute and were concatenated into 20 fractions by combining pre‐concatenation fractions 1, 21, 41, 61, and 81; 2, 22, 42, 62 and 82; and so on. Twenty fractions were made in this study because a balance was required in order to achieve high throughput and sensitivity of protein identification. A schematic of the proteomic workflow to discover significant differentially expressed proteins in patients with HF is shown in the online supplementary *Figure* *S1*.

### Liquid chromatography electrospray ionisation mass spectrometry/mass spectrometry analysis

A Waters Synapt G2 High Definition Mass Spectrometry (HDMS) system (Waters Corporation) was employed to analyse the HF samples in this study. This is a hybrid, quadrupole, ion mobility, orthogonal acceleration, time of flight mass spectrometer controlled by the MassLynx 4.1. A NanoLockSpray Electrospray Ionisation (ESI) source is fitted as standard equipment and a nanoAcquity Ultra Performance Liquid Chromatography (UPLC) system was coupled online. The sample was introduced into the source through an emitter with a flow rate of 0.3 μL/min.

Using the fractionation and concatenation method, each HF group had a total of 20 concatenated samples that were analysed on liquid chromatography electrospray ionisation mass spectrometry/mass spectrometry (LC ESI‐MS/MS) in ion mobility LC‐data independent acquisition mass spectrometry (HDMSE) mode. One μg of each fraction was analysed in each run. Fractions were analysed randomly and the same fraction of both HF groups was analysed at the same time. Each fraction was analysed in triplicate using a 50 min gradient in LC ESI‐MS/MS in HDMSE mode. The column was washed by three injections of a 20 min run (1 × 20% isopropanol, 1 × methanol, and 1 × 0.1% formic acid) between each triplicate injection to avoid carryover.

### Quality control

The use of a quality control (QC) is necessary in proteomic experiments for analysing samples over a long period of time in order to maintain a high standard of obtained results. At the same time of making two pooled HF plasma samples, a 10 μL aliquot of each plasma sample was also taken from 100 HF plasma samples and pooled together to make a QC plasma sample. This pooled QC sample was prepared and analysed throughout the whole experiment as the pooled HF samples. The QC sample was analysed in triplicate at the beginning of the experiment, then in the middle of the experiment, and at the end of the experiment on mass spectrometry along with the pooled HF plasma samples that allows the intra‐study assessment of data quality. The chromatography of every QC sample was assessed on MassLynx 4.1 and PLGS 2.5 processed the raw data. If the chromatography and the total number of proteins identified were similar to the first QC sample, then the next samples were analysed.

### Data analysis and statistics

Progenesis QI for Proteomics (Nonlinear Dynamics, Manchester, UK) enabled the identified and quantified proteins to be compared between two HF groups. The database was downloaded from UniProtKB database (Human) in FASTA format. Data processing parameters were minimum of two fragments per peptide, minimum of five fragments per protein, and minimum of two peptides per protein. The database was searched by using strict trypsin cleavage rules, two missed cleavages were allowed. Fixed modification of cysteine (Carbamidomethyl C) was selected. The variable modifications were Deamidation N, Oxidation M and Phosphoryl STY. The false rate discovery (FDR) for identification at the protein level was set to a maximum rate of 1%. Data generated using the Progenesis QI was exported to Microsoft Excel where further data analysis was performed. A multivariate statistical analysis was conducted on proteins and a further filter on proteins was applied based on the *P*‐value and fold change to identify the expression of proteins of interest.

The statistical software SPSS 24.0 (Statistical Package for the Social Sciences, Chicago, IL, USA) for Windows was employed for statistical analyses in this study. Values were compared by using Student's *t*‐tests for two group comparisons. All statistical tests were performed two‐tailed, and a *P*‐value <0.05 was considered statistically significant. Proteins with a fold change ≥2 and at a *P*‐value <0.05 were significant differentially expressed proteins.

ProteinCenter software (Thermo Scientific) was employed to annotate significant differentially expressed proteins identified using Gene Ontology (GO terms) in order to classify them into biological processes such as metabolic processes, cellular homeostasis and so on. Mapping of significant differentially expressed proteins and pathway analysis were performed using Kyoto Encyclopedia of Genes and Genomes (KEGG) pathway database. The protein–protein interaction network analysis obtained by using ClueGO plug‐in from Cytoscape version 3.6.1. Cytoscape is a bioinformatics platform that is able to visualise and analyse networks. With ClueGO plug‐in built in Cytoscape, GO terms and KEGG pathways are integrated into a functionally organised network that is able to compare the biological roles of significant differentially expressed proteins.

## Results

### Patient characteristics

Patient characteristics of the HF cohort in this study are described in *Table* 1. Both HF (death/rehospitalisation and no events) groups were matched for age (average age: 76.6 ± 8.1 years) and gender. In the HF patients with events, estimated glomerular filtration rate was lower (45.76 ± 14.23 mL/min^‐1^ vs. 51.34 ± 11.19 mL/min^‐1^; *P* = 0.037) and white blood cell count (1000/mm^3^) was higher (9.11 ± 3.10 vs. 7.97 ± 3.82; *P* = 0.016). More patients were in New York Heart Association (NYHA) class III/IV (76% and 54% for HF with death/rehospitalisation and no events, respectively; *P* = 0.021). In addition, waist‐to‐hip ratio was higher (1.01 ± 0.13 vs. 0.96 ± 0.10; *P* = 0.018) and more patients had medical history of coronary artery bypass surgery (18 vs. 6; *P* = 0.005) and peripheral arterial disease (21 vs. 8; *P* = 0.006) in patients with events. NT‐proBNP levels were higher in HF patients with events (6321.58 ± 7557.40 pg/mL vs. 2616.38 ± 3442.63 pg/mL; *P* = 0.003). All other patient characteristics were not significantly different between the two HF groups.

### Quantitative proteomics and identification of over‐ or under‐expressed proteins

**Figure 1:**
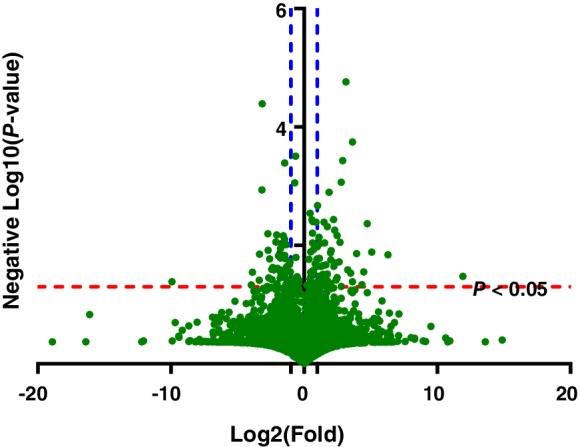
Volcano plot for proteins identified in both heart failure groups. The volcano plot displays proteins identified with differential expressions between both heart failure groups (death/rehospitalisation vs. no events). The horizontal axis (x‐axis) corresponds to the log 2 fold change value and the vertical axis (y‐axis) describes the negative value of log 10 (*P*‐value). The red dashed line represents *P* = 0.05 and the blue dashed lines represents the fold change = 2.

A total of 3001 quantified plasma proteins were identified in both HF groups that corresponded to a total of 57 718 peptides at FDR of 1% using two‐dimensional (2D) LC‐ESI‐MS/MS in HDMSE mode and label‐free quantification. There were 1426 up‐regulated proteins and 1562 down‐regulated proteins in the death/rehospitalisation HF group in comparisons with HF group with no events (*Figure* 1). In addition, there were 97 proteins identified in both HF groups that were over‐ or under‐expressed significantly with expression changes more than two‐fold at *P* < 0.05 in the comparison between the death/rehospitalisation HF group and HF group with no events, including 51 up‐regulated and 46 down‐regulated proteins in the death/rehospitalisation HF group (*Table* 2; online supplementary *Table S1* and *Figure S2*).

**Table 2:** List of significant differentially expressed proteins with a fold change more than 10 in comparisons between both heart failure groups

Protein name	Gene	Fold change	Expression	*P*‐value
Copine‐2	CPNE2	11.40	Up	0.0292
DnaJ homolog subfamily C member 7	DNAJC7	12.48	Up	0.0002
Dysferlin	DYSF	12.47	Up	0.0412
FERM domain‐containing protein 7	FRMD7	0.10	Down	0.0141
Matrix metalloproteinase 9	MMP9	0.06	Down	0.0464
Mucolipin‐3	MCOLN3	0.07	Down	0.0285
Nuclear pore complex protein Nup98‐Nup96	NUP98	20.43	Up	0.0462
Nucleolar RNA helicase 2	DDX21	15.30	Up	0.0137
Protein FAM49B	FAM49B	26.91	Up	0.0043
Protein phosphatase 1 regulatory subunit 14B	PPP1R14B	33.57	Up	0.0130
Regulator of G‐protein signalling 10	RGS10	0.10	Down	0.0386
Trafficking kinesin‐binding protein 1	TRAK1	22.27	Up	0.0281
Transcription elongation factor SPT4	SUPT4H1	79.28	Up	0.0144

Fold change: ratio of heart failure with death/rehospitalisation:heart failure with no events. The rest of significant differentially expressed proteins is presented in the online supplementary *Table S1*.

### Functional classification of significant differentially expressed proteins

**Figure 2:**
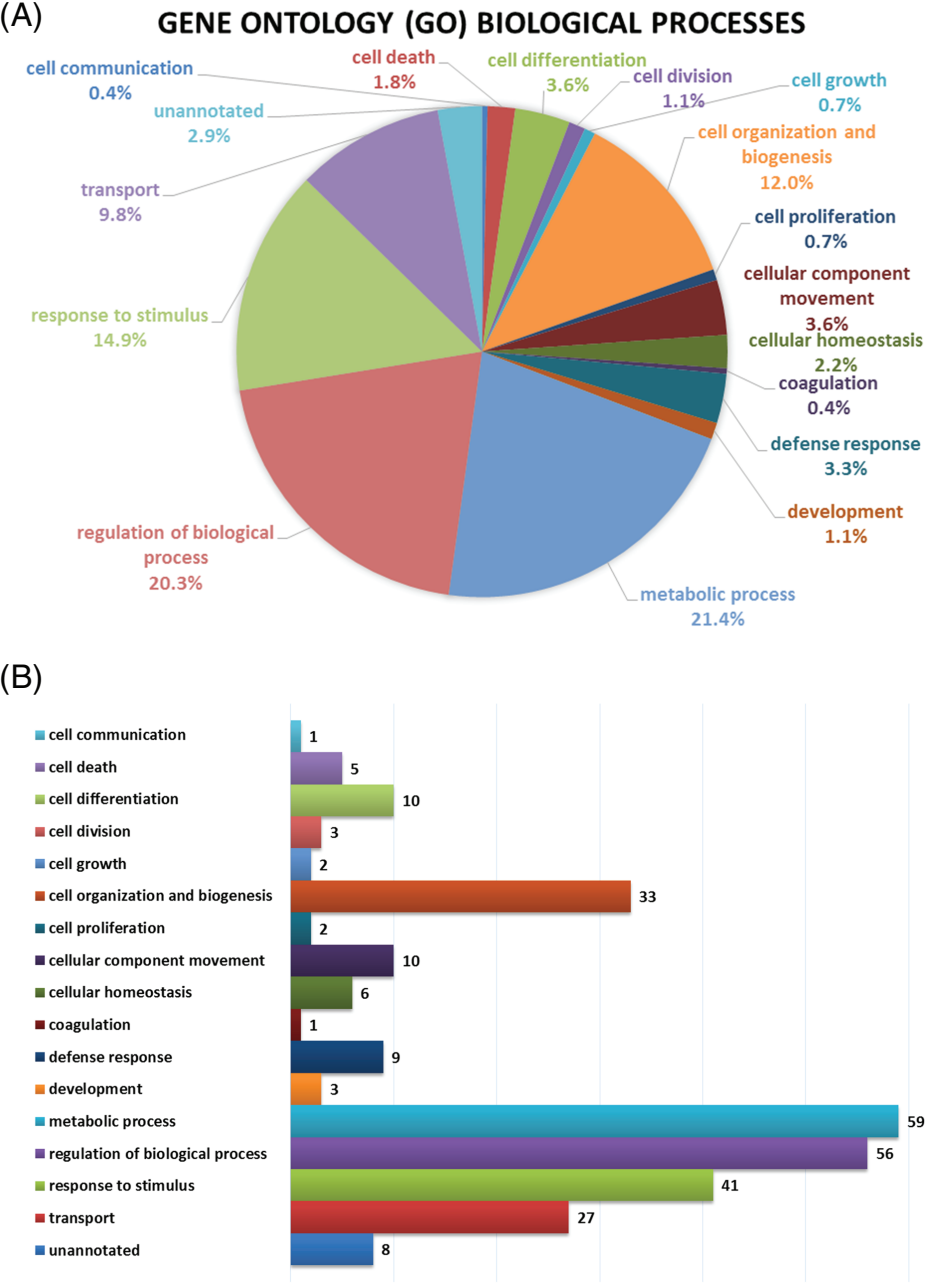
Gene ontology (GO) enrichment analysis of significant differentially expressed proteins in patients with heart failure with poor outcomes. (*A*) Proportions of significant differentially expressed proteins that were found in involving with biological processes. (*B*) Numbers of significant differentially expressed proteins that correspond to biological processes.

A GO analysis was performed in order to obtain a GO classification for the 97 significant differentially expressed proteins in patients with HF. The GO analysis of these proteins revealed involvement in multiple biological processes in HF. The highest proportions of 21.4% (59) proteins were found to be involved with metabolic processes in the biological processes of 97 significant differentially expressed proteins; 20.3% (56) proteins were related to the regulation of biological processes. The response to stimulus, cell organisation and biogenesis, and transport contributed to 14.9% (41), 12.0% (33) and 9.8% (27) proteins, respectively. The remaining proteins were involved with various biological processes such as cellular component movement (3.6%, 10 proteins), cell differentiation (3.6%, 10 proteins), defense response (3.3%, nine proteins), cellular homeostasis (2.2%, six proteins), cell death (1.8%, five proteins), cell division (1.1%, three proteins), development (1.1%, three proteins), cell growth (0.7%, two proteins), cell proliferation (0.7%, two proteins), coagulation (0.4%, one protein), and cell communication (0.4%, one protein). *Figure* 2 displays GO enrichment analysis of significant differentially expressed proteins in patients with HF.

### Protein–protein interaction analysis of significant differentially expressed proteins

**Figure 3:**
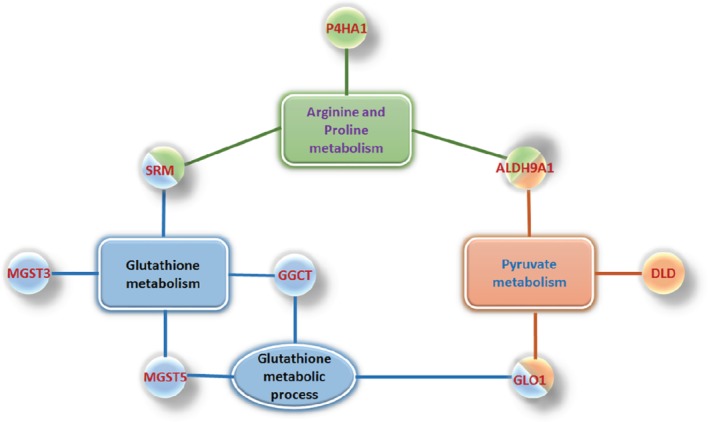
Protein–protein interaction networks of significant differentially expressed proteins revealed the central role of metabolism in poor clinical outcomes of patients with heart failure. A cluster of significant differentially expressed proteins is displayed that relates to glutathione metabolism, arginine and proline metabolism, and pyruvate metabolism in the pathogenesis of disease progression in heart failure and their involvement with poor clinical outcomes in patients with heart failure. The expression of these significant differentially expressed proteins is presented in more details in the online supplementary *Table S1*. ALDH9A1, 4‐trimethylaminobutyraldehyde dehydrogenase; DLD, dihydrolipoyl dehydrogenase mitochondrial; GGCT, gamma‐glutamylcyclotransferase; GLO1, lactoylglutathione lyase; GSTM5, glutathione S‐transferase Mu 5; MGST3, microsomal glutathione S‐transferase 3; P4HA1, prolyl 4‐hydroxylase subunit alpha‐1; SRM, spermidine synthase.

To explore the protein–protein interaction information of significant differentially expressed proteins the functional overview of 97 significant differentially expressed proteins was performed using Cytoscape software version 6.3.1 with ClueGO plug‐in. GO enrichment analysis and KEGG pathway of significant differentially expressed proteins revealed the central role of metabolic processes in clinical outcomes of patients with HF. The findings suggested that a cluster of proteins related to glutathione metabolism, arginine and proline metabolism, and pyruvate metabolism in the pathogenesis of poor outcome in patients with HF (*Figure* 3).

## Discussion

A total of 3001 proteins with quantification in both HF groups were identified using plasma proteome, with many of those over‐ or under‐expressed in the HF group with death/rehospitalisation as compared to the HF group with no events. We further explored the 97 proteins with a differential expression more than two‐fold at *P* < 0.05 in the comparison between two HF groups (death/rehospitalisation vs. no events). These results suggest that a wide range of proteins might be related to several pathophysiological processes in HF. Functional annotation of these results using GO, KEGG and protein–protein interaction networks revealed the complex functions of the proteomic interactome in the pathogenesis of poor outcome of patients with HF (*Figure* 2 and *Figure* 3). KEGG pathway analysis suggested that a cluster of significant differentially expressed proteins relates to glutathione metabolism, arginine and proline metabolism, and pyruvate metabolism in the pathogenesis of disease progression in HF and their involvement with clinical outcomes in patients with HF (*Figure* 3).

### Glutathione metabolism as a potential target for therapy in heart failure

The proteomic data in this study revealed the involvement in glutathione metabolism of differentially expressed proteins that includes gamma‐glutamylcyclotransferase (GGCT), lactoylglutathione lyase (GLO1), glutathione S‐transferase Mu 5 (GSTM5), microsomal glutathione S‐transferase 3 (MGST3), and spermidine synthase (SRM). The glutathione (L‐gamma‐glutamyl‐cysteinyl‐glycine) is an antioxidant that plays an important role in vascular and cardiac function^29^, ^30^ and determines cell survival.^31^, ^32^ Glutathione deficiency is involved with HF progression and cardiac remodelling in animal models and humans. Adamyl *et al*.^29^ found that the left ventricle of human failing heart was depleted in total glutathione by 54%. In addition, the authors demonstrated that there was a glutathione deficiency in the left ventricle in 2‐month post‐myocardial infarction rats with chronic HF. Interestingly, treatment with the glutathione precursor N‐acetylcysteine for 1 month normalised glutathione in the left ventricle, improved left ventricular contractile function, and lessened adverse left ventricular remodelling in 3‐month post‐myocardial infarction rats.^29^ Another study by Damy *et al*.^33^ discovered that glutathione deficiency was involved with the functional status and structural cardiac abnormalities of patients with cardiac diseases. The results from their study showed that compared to healthy controls, blood glutathione levels in patients with coronary artery disease were decreased by 21% in patients with NYHA class I and by 40% in patients with NYHA class II to IV. Moreover, blood glutathione levels in the atrial tissue were severely depleted by 58% in patients with NYHA class IV as compared to patients with NYHA class I.^33^ In a community‐based case‐control study (Hisayama Study), Shimizu *et al*.^34^ found that the frequency of diabetes and systolic and diastolic blood pressures reduced with elevating glutathione levels. In addition, there were significantly lower glutathione levels in the cerebral infarction and haemorrhage cases as compared to the corresponding control groups. The authors concluded that the reduction of glutathione levels in plasma may be an independent risk factor for the development of cardiovascular disease. Kovacs *et al*.^35^ investigated the link between myocardial infarction and glutathione S‐transferase polymorphism in patients that underwent cardiac surgery using genetic tests. The results suggested that the presence of allele B might have a potential role for protecting in the development of myocardial infarction, while the presence of alleles A and C could be associated with an increased risk of myocardial infarction. The findings about the involvement of differentially expressed proteins linked with glutathione metabolism in this study suggest that glutathione metabolism may be an interesting therapeutic target for HF.

### Arginine and proline metabolism as potential targets for therapy in heart failure

The proteomic results in this study found differentially expressed proteins such as 4‐trimethylaminobutyraldehyde dehydrogenase (ALDH9A1), prolyl 4‐hydroxylase subunit alpha‐1 (P4HA1) and SRM, which are responsible for arginine and proline metabolism. SRM also participates in glutathione metabolism (see above). Arginine is a precursor for the synthesis of proteins, nitric oxide, urea, polyamines, proline, glutamate, creatine, and agmatine^36^ and plays an important role in the oxidation of energy substrates in adipocytes, heart, liver, skeletal muscle, and other tissues. Arginine reverses endothelial dysfunction in patients with major cardiovascular risk factors (hypercholesterolaemia, smoking, hypertension, diabetes, obesity/insulin resistance, and aging) and improves cardiovascular disorders (coronary and peripheral arterial disease, ischaemia/reperfusion injury, and HF).^37^ HF is related to decreased plasma arginine levels. Investigating the effects of L‐arginine in chronic HF, Bocchi *et al*.^38^ found that L‐arginine decreased heart rate, mean systemic arterial pressure, systemic vascular resistance, and increased right atrial pressure, cardiac output, stroke volume. The authors concluded that L‐arginine improved cardiac function by reversing endothelial dysfunction with no effect on left ventricular contractility. Another study by Hambretch *et al*.^39^ also demonstrated that endothelial dysfunction in patients with HF can be improved by dietary supplementation with L‐arginine. Moreover, Rector *et al*.^40^ indicated that supplemental oral L‐arginine significantly increased forearm blood flow during forearm exercise, increased distances during a 6‐min walk test, lowered scores on the Living with HF questionnaire, improved arterial compliance, and reduced circulating levels of endothelin. Fan *et al*.^41^ demonstrated that arginine pathways in patients with acute myocardial infarction were activated as compared to unstable angina patients by using plasma metabolomic profiles. Furthermore, the pathway analysis by Wang *et al*.^42^ indicated that arginine and proline metabolism were affected in unstable angina patients complicated with diabetes. Therefore, arginine metabolism may be an interesting therapeutic target and supplementation of arginine could have beneficial effects in HF.

### Pyruvate metabolism as a potential target for therapy in heart failure

Pyruvate metabolism dysregulation was also suggested from the differentially expressed proteins in this study such as ALDH9A1 (shared with arginine and proline metabolism), dihydrolipoyl dehydrogenase mitochondrial (DLD) and GLO1 (shared with glutathione metabolism). Pyruvate is a keystone molecule for many aspects of metabolism in human. Pyruvate plays an important role for mitochondrial ATP generation in mitochondria by oxidative phosphorylation and for driving multiple biosynthetic pathways intersecting the citric acid cycle.^43^ Mutations of the genes encoding for proteins that regulate pyruvate metabolism may lead to many diseases. Pyruvate is an important source of energy for myocardium as an intermediate in Krebs cycle. Hermann *et al*.^44^ demonstrated that application of pyruvate into the left main coronary artery in patients with congestive HF with NYHA class III resulted in a 23% increase in cardiac index, a 38% increase in stroke volume index, a 36% decrease in pulmonary capillary wedge pressure, and heart rate decreased significantly by 11%. The authors suggest that the inotropic effect of pyruvate was associated with a significant decrease in heart rate in contrast to catecholamines and phosphodiesterase inhibitors. Another study showed that pyruvate increases the free energy from ATP hydrolysis and the sarcoplasmic reticulum (SR)‐calcium gradient.^45^ Moreover, Hasenfuss *et al*.^46^ indicate that application of pyruvate improves contractile performance of failing human myocardium by increasing intracellular Ca^2+^ transients and myofilament Ca^2+^ sensitivity. The effects of pyruvate on the SR calcium pump function are very interesting because SR calcium pump dysfunction has a pivotal role in the pathogenesis of HF.^47^ Fatima *et al*.^48^ demonstrated a disturbance of the pyruvate pathway in patients with coronary artery disease as compared to young healthy volunteers. Improving pyruvate availability could therefore be a novel approach to treat patients with HF.

Heart failure is a complex clinical syndrome at the end stage of heart disease that is comprised of multiple pathophysiological processes. The plasma is easily accessible for clinical use and the largest representation of the human proteome in which many proteins are related to pathogenesis for clinical applications. However, the greatest challenge in analysing plasma proteome is the wide dynamic range of protein concentrations.^8^ To the best of our knowledge, this is the first study that performed 2D LC ESI‐MS/MS in HDMSE combined with an immunodepletion method on a MARS 14 column along with using a sample pooling strategy for patients with HF. This proteomic pipeline was a reasonable approach that helped to overcome the low throughput and time‐consuming steps in mass spectrometry‐based proteomics and achieved a much greater identification of low abundant proteins in plasma. A wide range of differentially expressed proteins identified in this study not only provided more information about pathogenesis of disease progression in HF, but also discovered potential novel treatment targets for design of new drugs to improve poor outcomes in patients with HF. In addition, with this plasma proteomic approach a panel of multiple biomarkers in HF could be designed and developed that would be able to predict clinical events and treatment response with an improvement of precision and robustness because an ideal single biomarker may not exist for HF due to the participation of multiple pathogenic processes. Furthermore, these differentially expressed proteins identified could be employed to investigate pathogenic genomic loci in HF.

There was little literature about proteomic studies in patients with HF. In a recent proteomic study published by our group, 49 differently expressed proteins were identified between deaths (*n* = 45) and survivors (*n* = 45) in patients with HF. However, Emmens *et al*.^23^ focused on a depth investigation of the multifunctional HDL proteome to reveal underlying pathophysiological mechanisms that could explain the association between HDL and clinical outcome, while this study covered the whole plasma proteome in patients with HF. In another proteomic study by Stenemo *et al*.,^49^ 18 proteins were identified with several novel associations between proteins involved in apoptosis, inflammation, matrix remodelling, and fibrinolysis with incident HF. However, this study was performed in a HF cohort with elderly individuals and Proseek Multiplex cardiovascular disease I96 x 96 proximity extension assay technique was used to assess proteins previously associated with cardiovascular pathology. Farmakis *et al*.^50^ identified a set of 107 specific peptide biomarkers in HF with reduced ejection fraction (HFrEF) patients with chronic kidney disease (59 HFrEF patients vs. 67 controls). However, the capillary electrophoresis–mass spectrometry analysis was performed in this study using urine proteome.

### Future perspectives

The results in this study showed that plasma proteomics provides insights into the pathogenesis of disease progression in HF that would open new perspectives for translational research. The findings in this study also suggested that glutathione metabolism, arginine and proline metabolism, and pyruvate metabolism are involved in the progression of HF. They may be potential therapeutic targets that might provide potential additive therapeutic interventions for patients with HF. Therapies that modulate glutathione, arginine and pyruvate metabolism could have beneficial effects in HF by improving cardiac function and endothelial dysfunction that would be a topic for future investigation.

### Strengths and limitations

**Figure 4:**
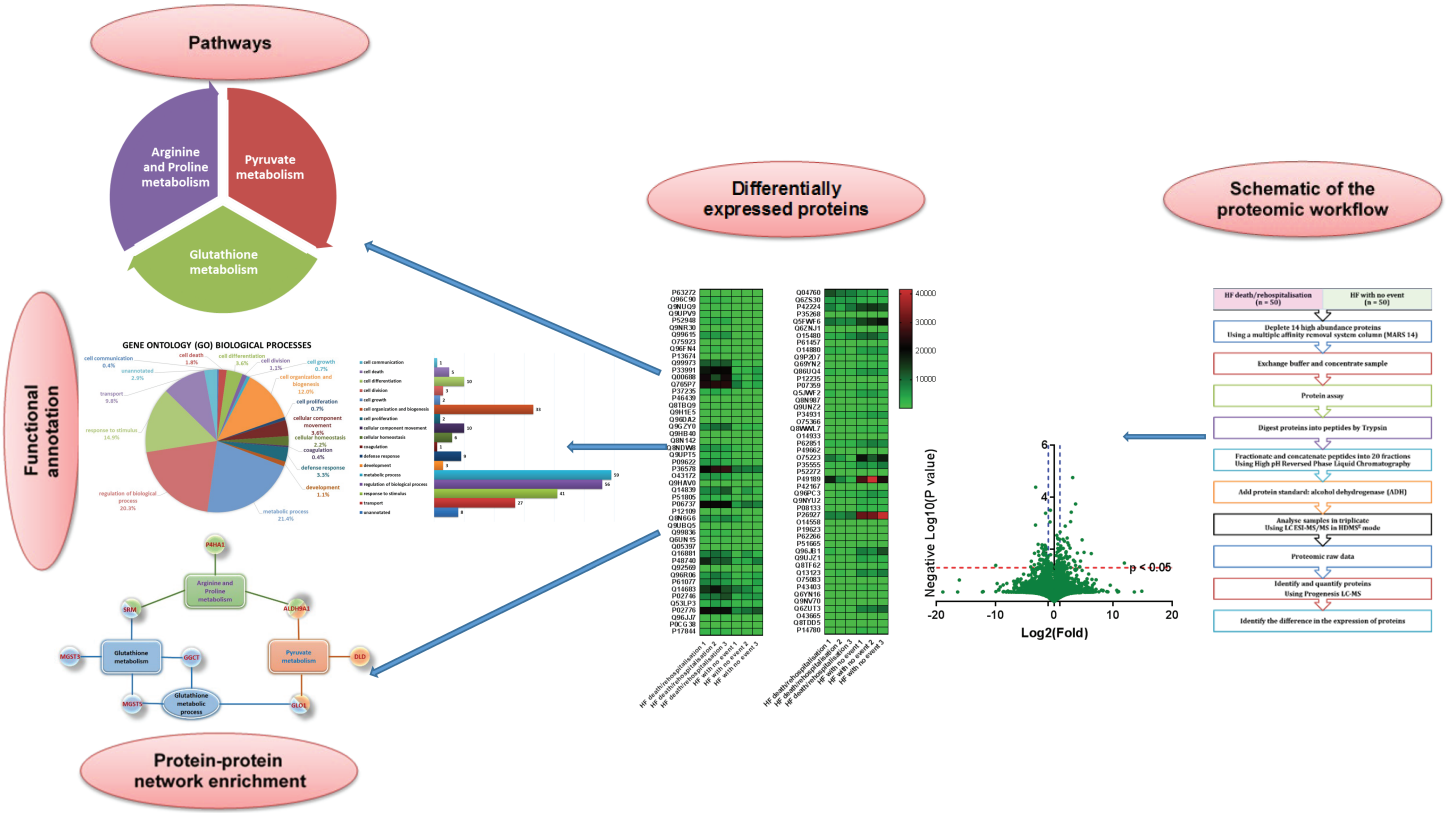
Schematic plasma proteomic approach to understand the pathogenesis and its link with clinical outcomes in patients with heart failure.

To the best of our knowledge, this is the first study using a plasma proteomic approach (2D LC ESI‐MS/MS in HDMSE) to increase the understanding about the pathogenesis of HF and its link with clinical outcomes in patients with HF. The results obtained in this study demonstrated that 2D LC ESI‐MS/MS in HDMSE combined with a multiple affinity removal system (MARS 14) is an informative feasible approach for providing insight into the pathogenesis of HF and discovery of potential biomarkers in plasma of patients with HF (*Figure* 4 *)*. Moreover, novel therapeutic targets could be identified for design of new drugs to improve poor outcomes in HF.

This study has several limitations. First, the sample size of this proteomic study was limited to 50 HF patients with death/rehospitalisation vs. 50 HF patients without events because these 100 patients with HF had the outcomes that were age‐ and sex‐matched at the time when the proteomic work was conducted. Therefore, the findings in this study will need to be validated in another large cohort. Second, sample pooling may lead to a loss of some inter‐individual biological information in plasma.^51^ However, pooling of samples in proteomics is a valid and valuable procedure,^52^ necessitated by the detailed analyses and large peptide/protein datasets in a low‐throughput workflow. Third, the method of immunoaffinity depletion for the top 14 high abundant proteins (MARS 14) in plasma was used to achieve a greater detection of low abundant proteins,^53^ but some nontargeted proteins might be removed along with the targeted proteins. Fourth, the differences of plasma proteomes between two subgroups (rehospitalisations vs deaths) in the group of HF patients with clinical events could not be analysed due to the procedure of sample pooling. Fifth, only Caucasian patients were recruited in the BIOSTAT‐CHF project due to the study design. Therefore, the results of this study may need replication in patients of non‐Caucasian ethnicity.

## Conclusions

From 3001 proteins identified with quantification, 97 were differentially expressed between HF patients with and without clinical events. A network analysis with pathway enrichment identified that pathways related to glutathione metabolism, arginine and proline metabolism, and pyruvate metabolism were activated in HF patients with clinical events. These findings imply that glutathione, arginine and pyruvate might be potential therapeutic targets to further improve clinical outcomes in HF.

## Funding

This work was funded by the European Union FP7 Project [FP7‐242209‐BIOSTAT‐CHF; EudraCT 2010‐020808‐29] and supported by the John and Lucille van Geest Foundation, the British Heart Foundation (FS/15/10/31223) and the National Institute for Health Research Leicester Biomedical Research Centre.

**Conflict of interest:** A.A.V. received consultancy fees and/or research grants from Amgen, AstraZeneca, Bayer, Boehringer Ingelheim, Cytokinetics, GSK, Myokardia, Novartis, Roche Diagnotics and Servier. S.D.A. reports grants from Vifor and Abbott Vascular, and fees for consultancy from Vifor, Bayer, Boehringer Ingelheim, Brahms, Janssen, Novartis, Servier, Stealth Peptides, and ZS Pharma. J.G.C. has received committee fees and/or research grants from Amgen, AstraZeneca, Bayer, Bristol‐Myers Squibb, GSK, Medtronic, Myokardia, Novartis, Philips, Pharmacosmos, PharmaNord, Sanofi, Servier, Stealth Biopharmaceuticals, Torrent Pharmaceuticals and Vifor. G.F. has received committee fees and/or research grants from Novartis, Bayer, Vifor, and Servier. C.C.L. has received consultancy fees and/or research grants from Amgen, AstraZeneca, Merck Sharp & Dohme, Novartis, and Servier. M.M. has received consulting honoraria from Amgen, AstraZeneca, Bayer, Novartis, Relypsa, Servier, Stealth Therapeutics, and Trevena; and speaker fees from Abbott Vascular and Servier. D.J.V.V. has received board membership fees or travel expenses from Novartis, and Johnson & Johnson. All other authors have reported that they have no relationships relevant to the contents of this paper to disclose.

## Supporting information


**Figure S1.** Schematic of the proteomic workflow to discover significant differentially expressed proteins in patients with heart failure.
**Figure S2.** Heatmap showing the expression profiles of 97 significant differentially expressed proteins in comparing between both heart failure groups.
**Table S1.** List of significant differentially expressed proteins with properties in patients with heart failure.

## References

[1] Cowie MR , Mosterd A , Wood DA , Deckers JW , Poole‐Wilson PA , Sutton GC , Grobbee DE . The epidemiology of heart failure. Eur Heart J 1997;18:208–225. 9043837 10.1093/oxfordjournals.eurheartj.a015223

[2] Senni M , Tribouilloy CM , Rodeheffer RJ , Jacobsen SJ , Evans JM , Bailey KR , Redfield MM . Congestive heart failure in the community: trends in incidence and survival in a 10‐year period. Arch Intern Med 1999;159:29–34. 9892327 10.1001/archinte.159.1.29

[3] Cowie MR , Wood DA , Coats AJ , Thompson SG , Suresh V , Poole‐Wilson PA , Sutton GC . Survival of patients with a new diagnosis of heart failure: a population based study. Heart 2000;83:505–510. 10768897 10.1136/heart.83.5.505 PMC1760808

[4] Stewart S , MacIntyre K , Hole DJ , Capewell S , McMurray JJ . More 'malignant' than cancer? Five‐year survival following a first admission for heart failure. Eur J Heart Fail 2001;3:315–322. 11378002 10.1016/s1388-9842(00)00141-0

[5] Levy D , Kenchaiah S , Larson MG , Benjamin EJ , Kupka MJ , Ho KK , Murabito JM , Vasan RS . Long‐term trends in the incidence of and survival with heart failure. N Engl J Med 2002;347:1397–1402. 12409541 10.1056/NEJMoa020265

[6] Mosterd A , Hoes AW . Clinical epidemiology of heart failure. Heart 2007;93:1137–1146. 17699180 10.1136/hrt.2003.025270 PMC1955040

[7] Askoxylakis V , Thieke C , Pleger S , Most P , Tanner J , Lindel K , Katus HA , Debus J , Bischof M . Long‐term survival of cancer patients compared to heart failure and stroke: a systematic review. BMC Cancer 2010;10:105. 20307299 10.1186/1471-2407-10-105 PMC2851688

[8] Anderson NL , Anderson NG . The human plasma proteome: history, character, and diagnostic prospects. Mol Cell Proteomics 2002;1:845–867. 12488461 10.1074/mcp.r200007-mcp200

[9] Qin S , Ferdinand AS , Richie JP , O'Leary MP , Mok SC , Liu BC . Chromatofocusing fractionation and two‐dimensional difference gel electrophoresis for low abundance serum proteins. Proteomics 2005;5:3183–3192. 16035113 10.1002/pmic.200401137

[10] Righetti PG , Boschetti E , Lomas L , Citterio A . Protein equalizer technology: the quest for a democratic proteome. Proteomics 2006;6:3980–3992. 16800034 10.1002/pmic.200500904

[11] Schiess R , Wollscheid B , Aebersold R . Targeted proteomic strategy for clinical biomarker discovery. Mol Oncol 2009;3:33–44. 19383365 10.1016/j.molonc.2008.12.001 PMC2753590

[12] Surinova S , Schiess R , Huttenhain R , Cerciello F , Wollscheid B , Aebersold R . On the development of plasma protein biomarkers. J Proteome Res 2011;10:5–16. 21142170 10.1021/pr1008515

[13] Ozturk K , Dow M , Carlin DE , Bejar R , Carter H . The emerging potential for network analysis to inform precision cancer medicine. J Mol Biol 2018;430:2875–2899. 29908887 10.1016/j.jmb.2018.06.016 PMC6097914

[14] Voors AA , Anker SD , Cleland JG , Dickstein K , Filippatos G , van der Harst P , Hillege HL , Lang CC , Ter Maaten JM , Ng L , Ponikowski P , Samani NJ , van Veldhuisen DJ , Zannad F , Zwinderman AH , Metra M . A systems BIOlogy Study to TAilored Treatment in Chronic Heart Failure: rationale, design, and baseline characteristics of BIOSTAT‐CHF. Eur J Heart Fail 2016;18:716–726. 27126231 10.1002/ejhf.531

[15] Ouwerkerk W , Voors AA , Anker SD , Cleland JG , Dickstein K , Filippatos G , van der Harst P , Hillege HL , Lang CC , Ter Maaten JM , Ng LL , Ponikowski P , Samani NJ , van Veldhuisen DJ , Zannad F , Metra M , Zwinderman AH . Determinants and clinical outcome of uptitration of ACE‐inhibitors and beta‐blockers in patients with heart failure: a prospective European study. Eur J Heart Fail 2017;38:1883–1890. 10.1093/eurheartj/ehx026 28329163

[16] Ferreira JP , Rossignol P , Machu J , Sharma A , Girerd N , Anker SD , Cleland JG , Dickstein K , Filippatos G , Hillege HL , Lang CC , Ter Maaten JM , Metra M , Ng L , Ponikowski P , Samani NJ , van Veldhuisen DJ , Zwinderman AH , Voors A , Zannad F . Mineralocorticoid receptor antagonist pattern of use in heart failure with reduced ejection fraction: findings from BIOSTAT‐CHF. Eur J Heart Fail 2017;19:1284–1293. 28580625 10.1002/ejhf.900

[17] Ouwerkerk W , Zwinderman AH , Ng LL , Demissei B , Hillege HL , Zannad F , van Veldhuisen DJ , Samani NJ , Ponikowski P , Metra M , Ter Maaten JM , Lang CC , van der Harst P , Filippatos G , Dickstein K , Cleland JG , Anker SD , Voors AA . Biomarker‐guided versus guideline‐based treatment of patients with heart failure: results from BIOSTAT‐CHF. J Am Coll Cardiol 2018;71:386–398. 29389354 10.1016/j.jacc.2017.11.041

[18] Tromp J , Westenbrink BD , Ouwerkerk W , van Veldhuisen DJ , Samani NJ , Ponikowski P , Metra M , Anker SD , Cleland JG , Dickstein K , Filippatos G , van der Harst P , Lang CC , Ng LL , Zannad F , Zwinderman AH , Hillege HL , van der Meer P , Voors AA . Identifying pathophysiological mechanisms in heart failure with reduced versus preserved ejection fraction. J Am Coll Cardiol 2018;72:1081–1090. 30165978 10.1016/j.jacc.2018.06.050

[19] Santema BT , Kloosterman M , Van Gelder IC , Mordi I , Lang CC , Lam CS , Anker SD , Cleland JG , Dickstein K , Filippatos G , Van der Harst P , Hillege HL , Ter Maaten JM , Metra M , Ng LL , Ponikowski P , Samani NJ , van Veldhuisen DJ , Zwinderman AH , Zannad F , Damman K , van der Meer P , Rienstra M , Voors AA . Comparing biomarker profiles of patients with heart failure: atrial fibrillation vs. sinus rhythm and reduced vs. preserved ejection fraction. Eur J Heart Fail 2018;39:3867–3875. 10.1093/eurheartj/ehy421 30137304

[20] Streng KW , Voors AA , Hillege HL , Anker SD , Cleland JG , Dickstein K , Filippatos G , Metra M , Ng LL , Ponikowski P , Samani NJ , van Veldhuisen DJ , Zwinderman AH , Zannad F , Damman K , van der Meer P , Lang CC . Waist‐to‐hip ratio and mortality in heart failure. Eur J Heart Fail 2018;20:1269–1277. 29963737 10.1002/ejhf.1244

[21] Beusekamp JC , Tromp J , van der Wal HH , Anker SD , Cleland JG , Dickstein K , Filippatos G , van der Harst P , Hillege HL , Lang CC , Metra M , Ng LL , Ponikowski P , Samani NJ , van Veldhuisen DJ , Zwinderman AH , Rossignol P , Zannad F , Voors AA , van der Meer P . Potassium and the use of renin–angiotensin–aldosterone system inhibitors in heart failure with reduced ejection fraction: data from BIOSTAT‐CHF. Eur J Heart Fail 2018;20:923–930. 29327797 10.1002/ejhf.1079

[22] Ter Maaten JM , Voors AA , Damman K , van der Meer P , Anker SD , Cleland JG , Dickstein K , Filippatos G , van der Harst P , Hillege HL , Lang CC , Metra M , Navis G , Ng L , Ouwerkerk W , Ponikowski P , Samani NJ , van Veldhuisen DJ , Zannad F , Zwinderman AH , de Borst MH . Fibroblast growth factor 23 is related to profiles indicating volume overload, poor therapy optimization and prognosis in patients with new‐onset and worsening heart failure. Int J Cardiol 2018;253:84–90. 29306478 10.1016/j.ijcard.2017.10.010

[23] Emmens JE , Jones DJL , Cao TH , Chan DCS , Romaine SPR , Quinn PA , Anker SD , Cleland JG , Dickstein K , Filippatos G , Hillege HL , Lang CC , Ponikowski P , Samani NJ , van Veldhuisen DJ , Zannad F , Zwinderman AH , Metra M , de Boer RA , Voors AA , Ng LL . Proteomic diversity of high‐density lipoprotein explains its association with clinical outcome in patients with heart failure. Eur J Heart Fail 2018;20:260–267. 29251807 10.1002/ejhf.1101

[24] Bayes‐Genis A , Nunez J , Zannad F , Ferreira JP , Anker SD , Cleland JG , Dickstein K , Filippatos G , Lang CC , Ng LL , Ponikowski P , Samani NJ , van Veldhuisen DJ , Zwinderman AH , Metra M , Lupon J , Voors AA . The PCSK9‐LDL receptor axis and outcomes in heart failure: BIOSTAT‐CHF subanalysis. J Am Coll Cardiol 2017;70:2128–2136. 29050560 10.1016/j.jacc.2017.08.057

[25] Ferreira JP , Rossignol P , Demissei B , Sharma A , Girerd N , Anker SD , Cleland JG , Dickstein K , Filippatos G , Hillege HL , Lang CC , Metra M , Ng LL , Ponikowski P , Samani NJ , van Veldhuisen DJ , Zwinderman AH , Voors A , Zannad F . Coronary angiography in worsening heart failure: determinants, findings and prognostic implications. Heart 2018;104:606. 28798192 10.1136/heartjnl-2017-311750

[26] Suzuki T , Yazaki Y , Voors AA , Jones DJL , Chan DC , Anker SD , Cleland JG , Dickstein K , Filippatos G , Hillege HL , Lang CC , Ponikowski P , Samani NJ , van Veldhuisen DJ , Zannad F , Zwinderman AH , Metra M , Ng LL . Association with outcomes and response to treatment of trimethylamine N‐oxide in heart failure: results from BIOSTAT‐CHF. Eur J Heart Fail 2019;21:877–886. 30370976 10.1002/ejhf.1338

[27] Cao TH , Jones DJL , Quinn PA , Chan DCS , Hafid N , Parry HM , Mohan M , Sandhu JK , Anker SD , Cleland JG , Dickstein K , Filippatos G , Hillege HL , Metra M , Ponikowski P , Samani NJ , van Veldhuisen DJ , Zannad F , Zwinderman AH , Voors AA , Lang CC , Ng LL . Using matrix assisted laser desorption ionisation mass spectrometry (MALDI‐MS) profiling in order to predict clinical outcomes of patients with heart failure. Clin Proteomics 2018;15:35. 30410428 10.1186/s12014-018-9213-1 PMC6214161

[28] Dickstein K , Cohen‐Solal A , Filippatos G , JJ MM , Ponikowski P , Poole‐Wilson P , Stromberg A , van Veldhuisen DJ , Atar D , Hoes AW , Keren A , Mebazaa A , Nieminen M , Priori SG , Swedberg K , Vahanian A , Camm J , de Caterina R , Dean V , Filippatos G , Funck‐Brentano C , Hellemans I , Kristensen SD , Mcgregor K , Sechtem U , Silber S , Tendera M , Widimsky P , Zamorano JL , Tendera M , Auricchio A , Bax J , Bohm M , Corra U , Della Bella P , Elliott PM , Follath F , Gheorghiade M , Hasin Y , Hernborg A , Jaarsma T , Komajda M , Kornowski R , Piepoli M , Prendergast B , Tavazzi L , Vachiery J , FW V , Zamorano JL , Zannad F . ESC Guidelines for the diagnosis and treatment of acute and chronic heart failure 2008. Eur Heart J 2008;29:2388–2442. 18799522 10.1093/eurheartj/ehn309

[29] Adamy C , Mulder P , Khouzami L , Andrieu‐abadie N , Defer N , Candiani G , Pavoine C , Caramelle P , Souktani R , Le Corvoisier P , Perier M , Kirsch M , Damy T , Berdeaux A , Levade T , Thuillez C , Hittinger L , Pecker F . Neutral sphingomyelinase inhibition participates to the benefits of N‐acetylcysteine treatment in post‐myocardial infarction failing heart rats. J Mol Cell Cardiol 2007;43:344–353. 17707397 10.1016/j.yjmcc.2007.06.010

[30] Yucel D , Aydogdu S , Cehreli S , Saydam G , Canatan H , Senes M , Cigdem Topkaya B , Nebioglu S . Increased oxidative stress in dilated cardiomyopathic heart failure. Clin Chem 1998;44:148–154. 9550572

[31] Haddad JJ , Harb HL . l‐γ‐Glutamyl‐l‐cysteinyl‐glycine (glutathione; GSH) and GSH‐related enzymes in the regulation of pro‐ and anti‐inflammatory cytokines: a signaling transcriptional scenario for redox(y) immunologic sensor(s)? Mol Immunol 2005;42:987–1014. 15829290 10.1016/j.molimm.2004.09.029

[32] Franco R , Schoneveld OJ , Pappa A , Panayiotidis MI . The central role of glutathione in the pathophysiology of human diseases. Arch Physiol Biochem 2007;113:234–258. 18158646 10.1080/13813450701661198

[33] Damy T , Kirsch M , Khouzami L , Caramelle P , Le Corvoisier P , Roudot‐Thoraval F , Dubois‐Randé JL , Hittinger L , Pavoine C , Pecker F . Glutathione deficiency in cardiac patients is related to the functional status and structural cardiac abnormalities. PLoS One 2009;4:e4871. 19319187 10.1371/journal.pone.0004871 PMC2655715

[34] Shimizu H , Kiyohara Y , Kato I , Kitazono T , Tanizaki Y , Kubo M , Ueno H , Ibayashi S , Fujishima M , Iida M . Relationship between plasma glutathione levels and cardiovascular disease in a defined population: the Hisayama study. Stroke 2004;35:2072–2077. 15256685 10.1161/01.STR.0000138022.86509.2d

[35] Kovacs V , Gasz B , Balatonyi B , Jaromi L , Kisfali P , Borsiczky B , Jancso G , Marczin N , Szabados S , Melegh B , Nasri A , Roth E . Polymorphisms in glutathione S‐transferase are risk factors for perioperative acute myocardial infarction after cardiac surgery: a preliminary study. Mol Cell Biochem 2014;389:79–84. 24435850 10.1007/s11010-013-1929-7

[36] Wu G , Morris SM . Arginine metabolism: nitric oxide and beyond. Biochem J 1998;336:1–17. 9806879 10.1042/bj3360001 PMC1219836

[37] Wu G , Meininger CJ . Arginine nutrition and cardiovascular function. J Nutr 2000;130:2626–2629. 11053497 10.1093/jn/130.11.2626

[38] Bocchi EA , Vilella de Moraes AV , Esteves‐Filho A , Fernando B , Auler JO , Carmona MJ , Bellotti G , Ramires AF . L‐Arginine reduces heart rate and improves hemodynamics in severe congestive heart failure. Clin Cardiol 2000;23:205–210. 10761810 10.1002/clc.4960230314 PMC6654780

[39] Hambrecht R , Hilbrich L , Erbs S , Gielen S , Fiehn E , Schoene N , Schuler G . Correction of endothelial dysfunction in chronic heart failure: additional effects of exercise training and oral L‐arginine supplementation. J Am Coll Cardiol 2000;35:706–713. 10716474 10.1016/s0735-1097(99)00602-6

[40] Rector TS , Bank AJ , Mullen KA , Tschumperlin LK , Sih R , Pillai K , Kubo SH . Randomized, double‐blind, placebo‐controlled study of supplemental oral l‐arginine in patients with heart failure. Circulation 1996;93:2135–2141. 8925582 10.1161/01.cir.93.12.2135

[41] Fan Y , Li Y , Chen Y , Zhao Y , Liu L , Li J , Wang SL , Alolga RN , Yin Y , Wang XM , Zhao DS , Shen JH , Meng FQ , Zhou X , Xu H , He GP , Lai MD , Li P , Zhu W , Qi LW . Comprehensive metabolomic characterization of coronary artery diseases. J Am Coll Cardiol 2016;68:1281–1293. 27634119 10.1016/j.jacc.2016.06.044

[42] Wang J , Xu W , Zhao H , Chen J , Zhu B , Li X , Deng D , Wang J , Liu J , Yu Y , Xiao H , Wang W . Identification of potential plasma biomarkers and metabolic dysfunction for unstable angina pectoris and its complication based on global metabolomics. Biosci Rep 2019;39:BSR20181658. 30770400 10.1042/BSR20181658 PMC6430740

[43] Gray LR , Tompkins SC , Taylor EB . Regulation of pyruvate metabolism and human disease. Cell Mol Life Sci 2014;71:2577–2604. 24363178 10.1007/s00018-013-1539-2 PMC4059968

[44] Hermann H , Pieske B , Schwarzmöller E , Keul J , Just H , Hasenfuss G . Haemodynamic effects of intracoronary pyruvate in patients with congestive heart failure: an open study. Lancet 1999;353:1321–1323. 10218531 10.1016/s0140-6736(98)06423-x

[45] Chen W , London R , Murphy E , Steenbergen C . Regulation of the Ca2+ gradient across the sarcoplasmic reticulum in perfused rabbit heart: a 19F nuclear magnetic resonance study. Circ Res 1998;83:898–907. 9797338 10.1161/01.res.83.9.898

[46] Hasenfuss G , Maier L , Hermann H , Luers C , Hunlich M , Zeitz O , Janssen PM , Pieske B . Influence of pyruvate on contractile performance and Ca(2+) cycling in isolated failing human myocardium. Circulation 2002;105:194–199. 11790700 10.1161/hc0202.102238

[47] Hasenfuss G , Reinecke H , Studer R , Meyer M , Pieske B , Holtz J , Holubarsch C , Posival H , Just H , Drexler H . Relation between myocardial function and expression of sarcoplasmic reticulum Ca2+‐ATPase in failing and nonfailing human myocardium. Circ Res 1994;75:434–442. 8062417 10.1161/01.res.75.3.434

[48] Fatima T , Hashmi S , Iqbal A , Siddiqui AJ , Sami SA , Basir N , Bokhari SS , Sharif H , Musharraf SG . Untargeted metabolomic analysis of coronary artery disease patients with diastolic dysfunction show disturbed oxidative pathway. Metabolomics 2019;15:98. 31236740 10.1007/s11306-019-1559-5

[49] Stenemo M , Nowak C , Byberg L , Sundström J , Giedraitis V , Lind L , Ingelsson E , Fall T , Arnloy J . Circulating proteins as predictors of incident heart failure in the elderly. Eur J Heart Fail 2018;20:55–62. 28967680 10.1002/ejhf.980

[50] Farmakis D , Koeck T , Mullen W , Parissis J , Gogas BD , Nikolaou M , Lekakis J , Mischak H , Filippatos G . Urine proteome analysis in heart failure with reduced ejection fraction complicated by chronic kidney disease: feasibility, and clinical and pathogenetic correlates. Eur J Heart Fail 2016;18:822–829. 27220540 10.1002/ejhf.544

[51] Zolg W . The proteomic search for diagnostic biomarkers: lost in translation? Mol Cell Proteomics 2006;5:1720–1726. 16546995 10.1074/mcp.R600001-MCP200

[52] Diz AP , Truebano M , Skibinski DO . The consequences of sample pooling in proteomics: an empirical study. Electrophoresis 2009;30:2967–2975. 19676090 10.1002/elps.200900210

[53] Hakimi A , Auluck J , Jones GDD , Ng LL , Jones DJL . Assessment of reproducibility in depletion and enrichment workflows for plasma proteomics using label‐free quantitative data‐independent LC‐MS. Proteomics 2014;14:4–13. 24167004 10.1002/pmic.201200563

