# Supplementary material for: Plasma proteomic approach in patients with heart failure: insights into pathogenesis of disease progression and potential novel treatment targets
Source: Eur J Heart Fail. 2019 Nov 6;22(1):70–80. doi: 10.1002/ejhf.1608 (PMC7028019; doi:10.1002/ejhf.1608)
Supplement: Supplementary file 1 — Figure S1. Schematic of the proteomic workflow to discover significant differentially expressed proteins in patients with heart failure. Figure S2. Heatmap showing the expression profiles of 97 significant differentially expressed proteins in comparing between both heart failure groups. Table S1. List of significant differentially expressed proteins with properties in patients with heart failure. [file EJHF-22-70-s001.docx]

**Supplementary data**

Supplementary Table 1: List of significant differentially expressed proteins with properties in patients with HF.

| Uniprot Accession | Protein Name | Gene | Peptide count | Unique peptide | Confidence score | Fold Change | Expression | P value |
| --- | --- | --- | --- | --- | --- | --- | --- | --- |
| P51665 | 26S proteasome non-ATPase regulatory subunit 7 | PSMD7 | 8 | 2 | 46 | 0.18 | Down | 0.0252 |
| P62266 | 40S ribosomal protein S23 | RPS23 | 4 | 2 | 27 | 0.19 | Down | 0.0378 |
| P62851 | 40S ribosomal protein S25 | RPS25 | 6 | 3 | 42 | 0.32 | Down | 0.0148 |
| P49189 | 4-trimethylaminobutyraldehyde dehydrogenase | ALDH9A1 | 15 | 7 | 86 | 0.29 | Down | 0.0351 |
| P35268 | 60S ribosomal protein L22 | RPL22 | 10 | 8 | 65 | 0.48 | Down | 0.0488 |
| P36578 | 60S ribosomal protein L4 | RPL4 | 25 | 17 | 137 | 4.00 | Up | 0.0438 |
| Q8N6G6 | ADAMTS-like protein 1 | ADAMTSL1 | 7 | 5 | 41 | 3.09 | Up | 0.0359 |
| Q8N142 | Adenylosuccinate synthetase isozyme 1 | ADSSL1 | 5 | 2 | 39 | 4.71 | Up | 0.0225 |
| P12235 | ADP/ATP translocase 1 | SLC25A4 | 16 | 3 | 116 | 0.39 | Down | 0.0457 |
| O75366 | Advillin | AVIL | 2 | 2 | 10 | 0.36 | Down | 0.0089 |
| Q53LP3 | Ankyrin repeat domain-containing protein SOWAHC | SOWAHC | 3 | 2 | 20 | 2.26 | Up | 0.0488 |
| P08133 | Annexin A6 | ANXA6 | 64 | 39 | 597 | 0.24 | Down | 0.0067 |
| Q96PC3 | AP-1 complex subunit sigma-3 | AP1S3 | 1 | 1 | 6 | 0.25 | Down | 0.0244 |
| Q86UQ4 | ATP-binding cassette sub-family A member 13 | ABCA13 | 44 | 14 | 218 | 0.39 | Down | 0.0097 |
| P49662 | Caspase-4 | CASP4 | 1 | 1 | 5 | 0.32 | Down | 0.0098 |
| Q14839 | Chromodomain-helicase-DNA-binding protein 4 | CHD4 | 15 | 6 | 84 | 3.35 | Up | 0.0397 |
| P12109 | Collagen alpha-1(VI) chain | COL6A1 | 35 | 25 | 298 | 3.10 | Up | 0.0213 |
| P02746 | Complement C1q subcomponent subunit B | C1QB | 8 | 4 | 55 | 2.29 | Up | 0.0443 |
| Q96FN4 | Copine-2 | CPNE2 | 9 | 2 | 50 | 11.40 | Up | 0.0292 |
| Q69YN2 | CWF19-like protein 1 | CWF19L1 | 3 | 1 | 19 | 0.40 | Down | 0.0228 |
| P09622 | Dihydrolipoyl dehydrogenase_ mitochondrial | DLD | 20 | 12 | 118 | 4.02 | Up | 0.0175 |
| P33991 | DNA replication licensing factor MCM4 | MCM4 | 13 | 8 | 91 | 7.51 | Up | 0.0004 |
| Q99615 | DnaJ homolog subfamily C member 7 | DNAJC7 | 3 | 1 | 16 | 12.48 | Up | 0.0002 |
| Q9P2D7 | Dynein heavy chain 1_ axonemal | DNAH1 | 37 | 11 | 190 | 0.40 | Down | 0.0373 |
| Q96JB1 | Dynein heavy chain 8_ axonemal | DNAH8 | 39 | 11 | 225 | 0.18 | Down | 0.0499 |
| O75923 | Dysferlin | DYSF | 18 | 6 | 95 | 12.47 | Up | 0.0412 |
| Q9UBQ5 | Eukaryotic translation initiation factor 3 subunit K | EIF3K | 4 | 2 | 24 | 3.05 | Up | 0.0424 |
| Q9NV70 | Exocyst complex component 1 | EXOC1 | 5 | 2 | 27 | 0.11 | Down | 0.0012 |
| Q9UPT5 | Exocyst complex component 7 | EXOC7 | 5 | 3 | 27 | 4.07 | Up | 0.0122 |
| Q6ZUT3 | FERM domain-containing protein 7 | FRMD7 | 5 | 3 | 28 | 0.10 | Down | 0.0141 |
| P35555 | Fibrillin-1 | FBN1 | 55 | 40 | 352 | 0.30 | Down | 0.0291 |
| Q05397 | Focal adhesion kinase 1 | PTK2 | 15 | 2 | 84 | 2.81 | Up | 0.0478 |
| Q8WWL7 | G2/mitotic-specific cyclin-B3 | CCNB3 | 9 | 5 | 42 | 0.35 | Down | 0.0332 |
| O75223 | Gamma-glutamylcyclotransferase | GGCT | 3 | 2 | 17 | 0.31 | Down | 0.0263 |
| P46439 | Glutathione S-transferase Mu 5 | GSTM5 | 9 | 4 | 55 | 6.24 | Up | 0.0190 |
| P06737 | Glycogen phosphorylase_ liver form | PYGL | 32 | 11 | 219 | 3.33 | Up | 0.0048 |
| Q5JWF2 | Guanine nucleotide-binding protein G(s) subunit alpha isoforms XLas | GNAS | 18 | 10 | 114 | 0.38 | Down | 0.0166 |
| Q9HAV0 | Guanine nucleotide-binding protein subunit beta-4 | GNB4 | 5 | 1 | 45 | 3.68 | Up | 0.0013 |
| P34931 | Heat shock 70 kDa protein 1-like | HSPA1L | 31 | 4 | 204 | 0.37 | Down | 0.0147 |
| O14558 | Heat shock protein beta-6 | HSPB6 | 6 | 4 | 64 | 0.21 | Down | 0.0259 |
| P26927 | Hepatocyte growth factor-like protein | MST1 | 20 | 9 | 106 | 0.23 | Down | 0.0087 |
| P52272 | Heterogeneous nuclear ribonucleoprotein M | HNRNPM | 43 | 30 | 366 | 0.30 | Down | 0.0112 |
| P37235 | Hippocalcin-like protein 1 | HPCAL1 | 9 | 4 | 59 | 6.45 | Up | 0.0089 |
| Q6YN16 | Hydroxysteroid dehydrogenase-like protein 2 | HSDL2 | 6 | 3 | 30 | 0.11 | Down | 0.0000 |
| Q04760 | Lactoylglutathione lyase | GLO1 | 12 | 10 | 100 | 2.17 | Up | 0.0448 |
| P42167 | Lamina-associated polypeptide 2_ isoforms beta/gamma | TMPO | 25 | 7 | 178 | 0.27 | Down | 0.0151 |
| P48740 | Mannan-binding lectin serine protease 1 | MASP1 | 31 | 3 | 210 | 2.65 | Up | 0.0309 |
| P14780 | Matrix metalloproteinase-9 | MMP9 | 2 | 1 | 9 | 0.06 | Down | 0.0464 |
| O15480 | Melanoma-associated antigen B3 | MAGEB3 | 6 | 4 | 36 | 0.46 | Down | 0.0418 |
| O14880 | Microsomal glutathione S-transferase 3 | MGST3 | 2 | 2 | 16 | 0.42 | Down | 0.0436 |
| Q765P7 | MTSS1-like protein | MTSS1L | 2 | 1 | 10 | 6.92 | Up | 0.0009 |
| Q8TDD5 | Mucolipin-3 | MCOLN3 | 3 | 2 | 22 | 0.07 | Down | 0.0285 |
| Q99836 | Myeloid differentiation primary response protein MyD88 | MYD88 | 4 | 2 | 20 | 2.94 | Up | 0.0091 |
| Q6ZS30 | Neurobeachin-like protein 1 | NBEAL1 | 11 | 5 | 65 | 2.02 | Up | 0.0021 |
| Q6ZNJ1 | Neurobeachin-like protein 2 | NBEAL2 | 7 | 3 | 37 | 0.47 | Down | 0.0211 |
| Q9UNZ2 | NSFL1 cofactor p47 | NSFL1C | 10 | 3 | 59 | 0.37 | Down | 0.0079 |
| Q8N987 | N-terminal EF-hand calcium-binding protein 1 | NECAB1 | 2 | 1 | 11 | 0.37 | Down | 0.0004 |
| P52948 | Nuclear pore complex protein Nup98-Nup96 | NUP98 | 8 | 2 | 60 | 20.43 | Up | 0.0462 |
| Q9GZY0 | Nuclear RNA export factor 2 | NXF2 | 8 | 2 | 40 | 5.06 | Up | 0.0198 |
| Q9NR30 | Nucleolar RNA helicase 2 | DDX21 | 18 | 8 | 111 | 15.30 | Up | 0.0137 |
| Q00688 | Peptidyl-prolyl cis-trans isomerase FKBP3 | FKBP3 | 12 | 5 | 90 | 6.92 | Up | 0.0493 |
| Q92569 | Phosphatidylinositol 3-kinase regulatory subunit gamma | PIK3R3 | 2 | 1 | 11 | 2.60 | Up | 0.0219 |
| P02776 | Platelet factor 4 | PF4 | 5 | 4 | 43 | 2.25 | Up | 0.0094 |
| P07359 | Platelet glycoprotein Ib alpha chain | GP1BA | 5 | 3 | 29 | 0.38 | Down | 0.0291 |
| P51805 | Plexin-A3 | PLXNA3 | 5 | 1 | 29 | 3.34 | Up | 0.0303 |
| P0CG38 | POTE ankyrin domain family member I | POTEI | 30 | 1 | 263 | 2.20 | Up | 0.0165 |
| Q6UN15 | Pre-mRNA 3'-end-processing factor FIP1 | FIP1L1 | 6 | 3 | 40 | 2.82 | Up | 0.0040 |
| P17844 | Probable ATP-dependent RNA helicase DDX5 | DDX5 | 36 | 12 | 291 | 2.18 | Up | 0.0073 |
| Q8TF62 | Probable phospholipid-transporting ATPase IM | ATP8B4 | 2 | 1 | 9 | 0.16 | Down | 0.0353 |
| P13674 | Prolyl 4-hydroxylase subunit alpha-1 | P4HA1 | 18 | 10 | 140 | 9.74 | Up | 0.0259 |
| Q96JJ7 | Protein disulfide-isomerase TMX3 | TMX3 | 8 | 3 | 59 | 2.23 | Up | 0.0328 |
| Q9NUQ9 | Protein FAM49B | FAM49B | 9 | 2 | 56 | 26.91 | Up | 0.0043 |
| Q8TBQ9 | Protein kish-A | TMEM167A | 2 | 2 | 14 | 5.60 | Up | 0.0068 |
| Q96C90 | Protein phosphatase 1 regulatory subunit 14B | PPP1R14B | 2 | 1 | 20 | 33.57 | Up | 0.0130 |
| Q13123 | Protein Red | IK | 10 | 5 | 65 | 0.15 | Down | 0.0120 |
| P61457 | Pterin-4-alpha-carbinolamine dehydratase | PCBD1 | 1 | 1 | 7 | 0.43 | Down | 0.0398 |
| Q96DA2 | Ras-related protein Rab-39B | RAB39B | 5 | 3 | 32 | 5.31 | Up | 0.0383 |
| O43665 | Regulator of G-protein signaling 10 | RGS10 | 4 | 3 | 31 | 0.10 | Down | 0.0386 |
| Q9HB40 | Retinoid-inducible serine carboxypeptidase | SCPEP1 | 4 | 2 | 24 | 4.95 | Up | 0.0055 |
| P42224 | Signal transducer and activator of transcription 1-alpha/beta | STAT1 | 33 | 22 | 271 | 0.49 | Down | 0.0281 |
| Q96R06 | Sperm-associated antigen 5 | SPAG5 | 2 | 2 | 8 | 2.53 | Up | 0.0426 |
| P19623 | Spermidine synthase | SRM | 11 | 6 | 71 | 0.20 | Down | 0.0237 |
| Q9UJZ1 | Stomatin-like protein 2_ mitochondrial | STOML2 | 15 | 8 | 114 | 0.17 | Down | 0.0169 |
| Q14683 | Structural maintenance of chromosomes protein 1A | SMC1A | 49 | 17 | 281 | 2.37 | Up | 0.0225 |
| Q99973 | Telomerase protein component 1 | TEP1 | 7 | 1 | 40 | 8.83 | Up | 0.0000 |
| Q8NDW8 | Tetratricopeptide repeat protein 21A | TTC21A | 9 | 1 | 44 | 4.65 | Up | 0.0037 |
| Q16881 | Thioredoxin reductase 1_ cytoplasmic | TXNRD1 | 32 | 13 | 236 | 2.75 | Up | 0.0423 |
| Q9H1E5 | Thioredoxin-related transmembrane protein 4 | TMX4 | 2 | 2 | 11 | 5.58 | Up | 0.0347 |
| Q9UPV9 | Trafficking kinesin-binding protein 1 | TRAK1 | 4 | 1 | 21 | 22.27 | Up | 0.0281 |
| P63272 | Transcription elongation factor SPT4 | SUPT4H1 | 1 | 1 | 5 | 79.28 | Up | 0.0144 |
| P43403 | Tyrosine-protein kinase ZAP-70 | ZAP70 | 4 | 1 | 28 | 0.15 | Down | 0.0494 |
| O43172 | U4/U6 small nuclear ribonucleoprotein Prp4 | PRPF4 | 3 | 2 | 16 | 3.94 | Up | 0.0132 |
| O14933 | Ubiquitin/ISG15-conjugating enzyme E2 L6 | UBE2L6 | 9 | 6 | 54 | 0.34 | Down | 0.0069 |
| P61077 | Ubiquitin-conjugating enzyme E2 D3 | UBE2D3 | 2 | 1 | 21 | 2.44 | Up | 0.0369 |
| Q9NYU2 | UDP-glucose:glycoprotein glucosyltransferase 1 | UGGT1 | 24 | 14 | 142 | 0.24 | Down | 0.0444 |
| O75083 | WD repeat-containing protein 1 | WDR1 | 32 | 17 | 253 | 0.15 | Down | 0.0063 |
| Q5FWF6 | Zinc finger protein 789 | ZNF789 | 4 | 2 | 21 | 0.48 | Down | 0.0191 |

**Fold change:** Ratio of HF with death/rehospitalisation: HF with no events.

**Supplementary Figure 1: Schematic of the proteomic workflow to discover significant differentially expressed proteins in patients with HF.**

| **Sample preparation** |  |
| --- | --- |
| **Sample analysis** |  |
| **Data analysis** |  |

**Supplementary Figure 2: Heatmap showing the expression profiles of 97 significant differentially expressed proteins in comparing between both HF groups (death/rehospitalisation vs no events) (Experiment performed in triplicate).**

Accession numbers corresponds to protein and gene names that are presented in *Supplementary Table 1*.
